# Related health risk assessment of exposure to arsenic and some heavy metals in gold mines in Banmauk Township, Myanmar

**DOI:** 10.1038/s41598-021-02171-9

**Published:** 2021-11-24

**Authors:** Pokkate Wongsasuluk, Aung Zaw Tun, Srilert Chotpantarat, Wattasit Siriwong

**Affiliations:** 1 College of Public Health Sciences, Chulalongkorn University, Bangkok, 10330 Thailand; 2 Health and Social Sciences and Addiction Research Unit (HSSRU), Chulalongkorn University, Bangkok, Thailand; 3 International Postgraduate Program in Hazardous Substance and Environmental Management, Chulalongkorn University, Bangkok, Thailand; 4 Center of Excellence on Hazardous Substance Management, Chulalongkorn University, Bangkok, Thailand; 5 Environmental Conservation Department, Office No. 58, Nay Pyi Taw, 15011 Myanmar; 6 Department of Geology, Faculty of Science, Chulalongkorn University, Bangkok, Thailand; 7 Research Unit of Green Mining (GMM), Chulalongkorn University, Bangkok, Thailand

**Keywords:** Environmental sciences, Environmental social sciences, Health occupations, Risk factors

## Abstract

Exposure to heavy metals in mining activities is a health issue among miners. This study was carried out at three small-scale gold mining sites situated in Banmauk Township, Myanmar and aims to assess the occupational health risks of small-scale gold miners who are exposed to arsenic (As), cadmium (Cd), mercury (Hg) and lead (Pb) in the soil through the dermal route. Soil samples were analyzed through atomic absorption spectroscopy (AAS). The concentrations of the heavy metals in soils found As, ranged 1.04 mg/kg to 22.17 mg/kg, 0.13 mg/kg to 3.07 mg/kg for Cd, 0.15 mg/kg to 77.44 mg/kg for Hg, and 7.67 mg/kg to 210.00 mg/kg for Pb. In this study, 79% of the participants did not use any form of personal protective equipment (PPE) while working in gold mining processes. Regarding noncancer risk assessment, the results found all hazard quotient were lower than acceptable level (HQ < 1). In addition, all hazard index (HI) was lover than 1, the highest HI was found as 5.66 × 10^−1^ in the amalgamation process. On the other hand, the result found cancer risk ranged from 8.02 × 10^−8^ to 1.75 × 10^−6^, and the estimated cancer risks for 9 years ranged from 4.78 × 10^−7^ to 1.04 × 10^−5^. Therefore, the cancer risks of the miners were greater than the United State Environmental Protection Agency (U.S. EPA) acceptable cancer risk level, 1 × 10^−6^, and the miners may be at risk of developing carcinogenic diseases. The suggestion is to educate miners about the health risks of heavy metals and to encourage the use of proper PPE all the time while working in gold mine.

## Introduction

Artisanal and small-scale mining (ASM) activities are widely conducted in many developing countries such as Central and South America, Asia, Africa and Oceania as these mining activities provide approximately three to five times more income than other rural-based small-scale agriculture, forestry and fishery activities^95^ . Gold mining is the most popular activity among the ASM activities^96^  . Although artisanal and small-scale gold mining (ASGM) activities are normally conducted under unsafe conditions, twenty to thirty percent of the total world gold supply is provided by ASGM^1^. These mining activities generally use simple tools and methods to extract gold from ore deposits ^96^. In these gold mining processes, mercury (Hg) is commonly used to capture gold; as a result, mercury can enter the environment in the soil, water, and atmosphere^2–5^. Therefore, ASGM has been identified as the world’s largest source of anthropogenic Hg released into the environment^6^. Furthermore, these mining activities can lead to increase the concentrations of heavy metals, such as arsenic (As), cadmium (Cd), Hg, lead (Pb), copper (Cu), and Zinc (Zn) which occur naturally in the Earth’s crust^7–14^. As a result, these increased concentrations of heavy metals can cause sediment, soil and water contamination, and many studies have reported carcinogenic and noncarnogenic diseases in humans because of exposure to heavy metals via inhalation, ingestion and dermal contact^15–22^.

Among the heavy metals, As, Cd, Hg, and Pb are classified as the heavy metals most toxic to humans and animals because of their elemental impurities, carcinogenicity and likelihood of occurrence^23–26^. Regarding the adverse health effects, the heavy metals can accumulate into target organs and tissues by carrying them through bloodstream, once it is taken up into the body and then can result serious health problems depending on lifestyle, demographic factors, duration, and magnitude of exposure. Exposure to As can cause a wide range of health problem ranging from gastrointestinal disturbance to development of neoplasms, anorexia, fever, fluid loss, goiter, hair loss, headache, herpes, impaired healing, muscle spasms, sore throat, weakness, especially liver, kidney and lymphatic cancer. Even low-level exposure to As can result abnormal heartbeat, blood vessels damage, abdominal pain, nausea and vomiting, and pricking sensations in the hands and legs in short term exposure, and long term exposure can leads to skin lesion, diabetes mellitus, pulmonary disease and cardiovascular disease, peripheral vascular disease, neurological problem, and certain type of cancers^27,28^. In case of exposure to Cd, health problem can range from aching pain in back and limb, muscular pain, chills, sweating, weakness, fever, and cancer. Long term exposure to Cd can cause fragile bone, lung damage, cardiovascular disease, diabetes, especially kidney and liver disease. It can also affects to brain, central nervous system, skeletal system, lung, stomach, heart, placenta, and female reproductive system^27,29^. Hg, which is non-essential and very toxic to human body, is mainly released from the anthropogenic activities like mining. Long term exposure to Hg can lead to serious effect on kidney and nervous system, and health problems can also hair loss, blisters on skin, rashes, nausea, diarrhea, joint pain, irritability and damaging stomach and intestines^30,31^. Regarding exposure to Pb, the sensitive targets are hematological and cardiovascular system, nervous system, and kidney. Long term exposure to Pb can result vomiting, forgetfulness, poor attention, coma, and death. Especially, it can cause serious health effect such as behavioral problem, learning disability, and death on young children^27,32,33^.

Furthermore, many studies have reported high concentrations of As, Cd, Hg and Pb in the soil and tailings of gold mining areas of Ghana^34,35^, Tanzania^36^, Iran^37^, Kenya^38,39^, Thailand^11,40,41^ and Oman^42^. Additionally, the main exposure pathway to these heavy metals from tailings and the soil to humans was reported to be the dermal route^43^. Exposure to these heavy metals can cause many serious health problems such as kidney and liver cancers, dermal keratosis^44^, damage to the central nervous system, heart disease^29^, joint pain, shyness, irritability^30^, vomiting, poor attention span, and headache^32,33^. Therefore, previous studies have conducted noncancer and cancer risk assessments of dermal exposure to these heavy metals in gold mining areas in Nigeria^45^, Ghana^46^, South Africa^47,48^, and China^43,49^, and unacceptable noncancer and cancer risks have been reported.

Gold mining activities have been conducted all over the country in Myanmar, especially in the Kachin, Sagaing, Mandalay, and Bago regions of Myanmar^50^. Therefore, Myanmar gold production was recorded as 1692 kg in 2015 and 1700 kg in 2016, and ASGM is the main sector of Myanmar’s gold production^51^. The ASGM activities also provide the great likelihoods to the local people for their socio-economy. Although gold mining activities are being widely conducted throughout in the country, a limited number of scientific papers exist regarding the human health risks of these gold mining activities. Few reports contain valuable information on small-scale gold mining in Kachin State of Myanmar but not including scientific data, were reported by Images Asia and Pan Kachin Development Society^52^ and Images Asia and Kachin Development Networking Group^53^. Earth Right International and Karen Environmental and Social Action Network additionally reported important information about gold mining in Shwekyin, Bago Region of Myanmar^54^, but it was not scientifically information. Though, Osawa & Hatsukawa^50^ published a scientific paper mentioned that environment of Myanmar might be contaminated with Hg released from ASGM. Kawakami et al.^55^ reported a high concentration of Hg in the atmosphere of ASGM area in Thabeikkyin Township, Mandalay Division of Myanmar. None of these previous studies conducted proper health risk assessments for miners. However, a recent research paper conducting health risk assessment by analyzing hair samples of the miners in the Thabeikkyin Township reported that neurological signs and symptoms of chronic Hg intoxication was investigated in 3 out of 18 miners^56^.

As mentioned, Myanmar still has limited information on gold mining impacts on miners especially in local small-scale mine. In addition, there is lack of information on health risk assessment that is conducting for specific exposure routes and environmental exposure such as air, water, and soil in terms of occupational health risks on miners. To fill these gaps, this study attempted to identify the local small-scale gold mining processes that might have adverse health effects on miners. And then, this study aims to assess carcinogenic and noncarcinogenic risks on both male and female miners who were exposed to the toxic metals such as As, Cd, Hg and Pb from their works.

This paper is the first health risk assessment report that is conducting on a specific exposure pathway to heavy metals existing in the environmental medium in terms of occupational health risk on miners in gold mine in Myanmar. This paper provides information on small-scale gold mining process, skin surface area of the miners and their job responsibilities, use of PPE of the miners, and conditions on carcinogenic and noncarcinogenic risks of miners.

## Small-scale gold mining in Myanmar

In Myanmar, the small-scale gold mining method involves the main steps of extraction of material, ore processing, concentration: sluicing and panning, amalgamation, and burning of the amalgam^3,50,57^.

In the extraction of the material, ore is excavated with backhoes after removing the topsoil that covers the ore deposits. Then, in the ore processing step, water is added into the excavated ore to form a slurry to separate gold and other material. The separated gold ore is then concentrated through gravity methods called sluicing and panning to further liberate the gold from other materials. The sluicing step uses the waterproof carpets that are placed over the surface of the wooden sluices to recover gold from the gold ore. Then, gold from the gold ore is further recovered by using a pan in the panning step. Then, Hg is mixed with the gold ore in a pan to obtain gold-Hg amalgam that is then squeezed by hand in a cloth bag. Then, the gold-Hg amalgam is heated to recover gold by evaporating Hg^3,4^. Finally, ore residues that have high amounts of Hg are removed, however, these Hg-rich ore residues are reprocessed again and again through the cyanide leaching method to further recover gold at some mining sites.

Among these gold mining steps, the extraction of the material step widely uses machines instead of human workers to remove the topsoil and to mine the ore, and human workers are mainly used in the other gold mining steps. However, at the step of burning the amalgam, miners can only be exposed to Hg vapor via the inhalation route, as this step is carried out in a particular place. As a result, among the gold mining steps, ore processing, sluicing, panning and amalgamation steps use mainly manual labor, and these gold mining steps are likely to include exposure to heavy metals in the soil on the body parts of the laborer through the dermal route. Therefore, these aforementioned four small-scale gold mining steps are the focus of occupational health risk assessments of miners in this study.

## Materials and methods

### The study area

The small-scale gold mining sites, namely, Sites A, B and C, near Nar Nant Htun Village in Banmauk Township, Sagaing Region, Myanmar, were the sites selected for this study. The areas permitted by the Government of Myanmar for Sites A, B and C are 0.081 km^2^, 0.081 km^2^ and 0.055 km^2^, respectively. In the permitted areas, the actual working area for Site A is 0.032 km^2^, Site B is 0.028 km^2^, and Site C is 0.016 km^2^. Normally, these mining sites are permitted by the government to conduct gold mining activities at their respective mining sites for one year. Therefore, after every year of gold mining, the site owners have to request a permit for the extension of their mining activities at the same sites for another year from the government. In this way, gold mining activities have been conducted at the same sites year by year. Therefore, at the same gold mining sites, Site A has been working for approximately five years, Site B has been working for approximately three years, and Site C has been working for approximately three years. These mining sites are situated approximately 46.7 km away from northeastern Banmauk town. Banmauk Township is one of the main gold mining areas in the Sagaing Region of Myanmar, and both legal and illegal gold mining activities can be widely found in Banmauk Township because of the wide occurrence of placer deposits in this area. However, research papers regarding the impacts of mining activities in this area are very limited. Placer deposits, which are very common for ASGM miners due to their ease of access and extraction of placer gold, can also be widely found in the vicinity of the study area.

### Data collection

This study was a cross-sectional study. Questionnaires and soil samples were collected in this study to investigate the occupational health risks of both male and female miners. This study has already been approved by The Ethics Review Committee for Research Involving Human Research Subjects, Health Science Group, Chulalongkorn University with certificate of approval—COA No. 002/2019 (Date of approval: 3 January 2019). Both the questionnaires and soil samples were collected in January 2019.

### Questionnaire survey and analysis

The targeted population in this study was both male and female small-scale gold miners within the age range of 18–60 years. Furthermore, miners were considered the targeted population only if they were working in the aforementioned gold mining steps and had a high potential for exposure to heavy metals in soil for at least 12 months. After recruiting the participants, a total of 53 participants (19 participants from site A, 17 participants from site B, and 17 participants from site C) were clearly explained the purpose of the study before the questions were asked. Continuously, information sheets that were written in English and Myanmar separately, including informed consent forms, were provided to the participants, and then they were asked to sign informed consent forms with full understanding and willingness. Then, face-to-face questions regarding the personal information of the miners such as height, weight, job responsibilities and personal protective equipment (PPE) use behavior were asked of the participants to prevent misunderstanding of the questionnaire, thereby obtaining information on the working behaviors of the miners in the gold mining steps.

### Soil samples collection

Three soil samples were collected from each gold mining step of ore processing, sluicing, panning, and amalgamation in each gold mining site in the study area. Therefore, a total of 36 soil samples were collected from 3 mining sites by using a steel trowel at a depth of 0–10 cm. To obtain a homogeneous soil sample, a 2 m × 2 m plot was created at each sampling point, and then five replicate soil samples were collected at the corners and center of each plot. Then, a 50-g homogeneous soil sample was finally collected from the bucket^58^. The collected soil samples were then put into polyethylene plastic bags that were thoroughly prewashed in 10% nitric acid and distilled water before use^59,60^. After collecting all samples, these samples were stored in an icebox and sent to the laboratory to analyze the concentrations of the aforementioned heavy metals and soil pH as soon as possible.

### Soil sample analysis

The collected soil samples were analyzed at the laboratory of the Department of Agriculture, Ministry of Agriculture, Livestock and Irrigation, Myanmar. To obtain a homogeneous sample, the soil samples were air dried first for two to three days and then sieved via a 2-mm nylon mesh. Then, each of the 5-g dry weight fine powder soil samples and 21-ml concentrated hydrochloric acid were mixed in a 250-ml beaker and swirled. Seven milliliters of concentrated nitric acid was also added to the mixture and swirled well again. Hence, the soil samples were digested on a hot plate at 115 °C in a fume cupboard for approximately 1–2 h for the dissolution of the heavy metals. The digested soil samples were then allowed to cool for approximately 20 min and filtered through Whatman No. 40 filter paper. Then, the filtrates were washed with hot distilled water, and they were collected and brought to a volume in a 100-ml volumetric flask with distilled water. This volume was then used for determination of the concentrations of heavy metals through AAS. The concentrations of Pb and Cd were determined by Agilent 240FS AA. However, Agilent 240FS AA with graphite tube atomizer (GTA) 120 was used for analyzing As, and Agilent 240FS AA with Agilent vapor generation accessory (VGA) 77 was applied for analyzing Hg.

Soil pH was measured by a Thermo Scientific™ Orion™ Star A211 pH benchtop meter with a soil:water ratio of 1:2.5 in the laboratory. Calibration of the pH meter was conducted after measuring every 10 soil samples by using pH buffers at 4, 7 and 10.

### Method validation and quality control

In this study, reagents used in the digestion process were AnalaR grade and met the requirements of the American Chemical Society Committee on Analytical Reagents. The calibration for quantification was automatically conducted by AAS. The digested samples were introduced into the AAS by an autosampler, and the wavelength was 193.3 nm. For the validation of the analytical method, validation parameters such as the limit of detection (LOD), limit of quantification (LOQ), precision and accuracy studies were conducted^61,62^.

#### Limit of detection (LOD)

The minimum concentration of analyte that can be detected but not necessarily quantified with an acceptable uncertainty is LOD. The LODs for each metal were determined as shown in Eq. (1) ^61,62^.

1 $$\text{LOD }=3\times {\text{S}}_{\text{bl}}, \quad $$

where S_bl_ is the standard deviation of the method blank.

#### Limit of quantification (LOQ)

The lowest concentration of an analyte in a sample that can be quantitatively determined with acceptable uncertainty is LOQ. LOQs for each metal were determined as shown in Eq. (2) ^61,62^.

2 $$\text{LOQ }=10\times {\text{S}}_{\text{bl}}, \quad $$

where S_bl_ is the standard deviation of the method blank.

#### Precision of the results

To determine the precision of the result, the relative standard deviations (RSDs) of the samples were assessed by spiking each sample in replicates of three at near mid-range calibration concentration. Afterwards, the replicated spiked samples were analyzed by applying the same procedure of the soil sample analysis. RSDs of the sample were calculated as shown in Eq. (3) ^61,62^.

3 $$\text{\%RSD}=\frac{\text{Standard Deviation}}{\text{Mean Value}} \times 100, $$

#### Result of quality control and assurance

The coefficients of the determination (r^2^) were ≥ 0.999 as shown in Supplementary Table 1, that were greater than the acceptable limit of 0.998 for the linearity of the regression line^62^. Thus, calibration curves for all the metals had good linearity with r^2^ indicating a good correlation between concentration and absorbance which means good calibration of the instrument. All the results of the heavy metals analysis in this study were above LODs; hence, the results of the analysis could be reliable. The RSDs values of the metals were also under the required control limits ≤ 15%^47,62^, which indicates the proposed method was precise and accurate.

### Ethics approval

This study has already approved by The Ethic Review Committee for Research Involving Human Research Subjects, Health Science Group, Chulalongkorn University with certificate of approval—COA No. 002/2019.

### Consent to participate

Informed consent was obtained from all individual participants included in the study.

## Health risk assessment

Risk assessment is generally conducted by following the four fundamental steps: hazard identification; dose–response assessment; exposure assessment; and risk characterization^63–65^.

### Hazard identification

Hazard identification seeks to qualitatively identify and review any potential incidence or degree of adverse health effects of a chemical substance by considering the exposure conditions^63–65^.

### Dose–response assessment

Dose–response assessment is the process that quantitatively estimates a relationship between the amount of exposure to a substance and the possibility of adverse health effects or disease^63,64,66^. Dose response is generally carried out by use of in vitro tests, in silico studies and studies in animals, particularly in rodents and in other species for data to apply to humans^67^. The suitable toxicity values that are used to evaluate the degree of adverse effects that occur in populations at risk for different exposure levels are developed from the quantitative dose–response relationship^63^. In this study, the cancer slope factor (CSF) and reference dose (RfD) of heavy metals for the dermal exposure route provided by the U.S. EPA was used in the evaluation of risks, the cancer slope factor for As was 1.5 (mg/kg/day)^−1^, and the RfDs for As, Cd, Hg, and Pb were 0.0003, 0.001, 0.0007, and 0.0036 (mg/kg-day), respectively^68^.

### Exposure assessment

Exposure assessment is defined as “the identification and evaluation of the human population exposed to a toxic agent, describing its composition and size, as well as the type, magnitude, frequency, route, and duration of exposure”^69,70^. The exposure assessment in this study was an occupational setting involving small-scale gold miners. The small-scale gold miners in the study area had the potential to be exposed to the aforementioned heavy metals in soil while working in the steps of ore processing, sluicing, panning, and amalgamation. Therefore, this study conducted cancer and noncancer risk assessments of dermal exposure to As, Cd, Hg and Pb in soils. Average daily intakes for the dermal route (ADI_dermal_) were calculated by using Eq. (4) provided by the USEPA^68^.

4 $${\text{ADI}}_{dermal} = \frac{\left({C}_{s}\times CF\times SA\times AF\times ABS\times EF\times ED\right)}{\left(BW\times AT\right)}, $$

where ADI_dermal_ is the average daily intake (mg/kg/day); C_S_ is the chemical concentration in soil (mg/kg); CF is the Conversation factor (kg/mg); SA is the skin surface area available for contact (cm^2^/day); AF is the soil adherence factor (mg/cm^2^); ABS is the absorption factor (unitless); EF is the exposure frequency (days/year); ED is the exposure duration (years); BW is the body weight (kg); and AT is the averaging time (days)^93,94^. Supplementary Table 2 shows the exposure parameters used in the calculation of average daily intake for dermal exposure (ADI_dermal_) in this study.

For skin surface area, the USEPA provided the total body surface area of men and women as 1.94 ± 0.00374 m^2^ and 1.69 ± 0.00374 m^2^, respectively, by studying Caucasian adults^71^, but the total body surface area of Myanmar people may not be the same as the total body surface area of Caucasian people. Therefore, the total body surface area of Myanmar people was calculated by using the weight and height of 42 male miners and 11 female miners through Eq. (5) ^72^.

5 $$\text{In TSA }=\text{ In }{a}_{0}+{a}_{1}\text{In H}+{a}_{2}\text{In W}, $$

where TSA is the total surface area in square meters; H is the height in centimeters; and W is the weight in kilograms; a_0_ = 0.024264; a_1_ = 0.3964; a_2_ = 0.5378.

### Risk characterization

Risk characterization summarizes and integrates the findings of the previous three steps of the risk assessment to qualitatively and/or quantitatively define the risk levels^63–65^. In this study, risk characterization predicted the potential carcinogenic and noncarcinogenic risks of small-scale gold miners who were dermally exposed to heavy metals in soil during working.

### Noncarcinogenic risk characterization

Noncarcinogenic health risks of small-scale gold miners who were exposed to As, Cd, Hg and Pb in soil via the dermal route were expressed as the hazard quotient (HQ). The hazard quotients for an individual heavy metal were calculated by using Eq. (6):

6 $$\text{Hazard Quotients }(\text{HQ}) = \frac{{{\text{ADI}}}}{{{\text{RfD}}}}, \quad \left(\text{USEPA},1991\right)$$

where HQ is the hazard quotient; ADI is the average daily dose (mg/kg/day); and RfD is the reference dose. After HQ was calculated, the hazard index (HI) was calculated by using Eq. (7).

7 $$\text{Hazard Index }\left(\text{HI}\right) = \sum \text{HQi, } \quad \left(\text{USEPA}, 1991\right)$$

where HI is the the sum of hazard quotients and HQi is the summation of HQ of noncarcinogens.

The RfD is an estimation of daily dermal exposure to the human population that is possible without a significant risk of harmful effects during a lifetime, and the hazard quotient (HQ) is an indicator of risks associated with health effects. The sum of the HQs is called the hazard index (HI), which assumes that the effects of the different compounds and effects are additive. If HI is greater than 1, there may be potential adverse systemic health concerns in exposed individuals. If HI is less than or equal to 1, there may be no significant adverse health effects^73,74^.

### Carcinogenic risk characterization

In this study, only arsenic (As) was considered for the cancer risks of dermal exposure to soil because cancer slope factors for the dermal route of the other heavy metals mentioned in this study were not available. Cancer risks were calculated by using the following Eq. (8):

8 $$\text{Cancer Risk }=\text{ ADI x CSF, } \quad \left(\text{USEPA}, 1991\right)$$

where CSF is the cancer slope factor (mg/kg/day)^−1^ and ADI is the average daily intake (mg/kg/day).

According to the U.S. EPA, the acceptable range for lifetime cancer risk is expressed as 1 × 10^–4^ to 1 × 10^–6^, that is, one in ten thousand to one in one million excess cancer cases. However, 1 × 10^–6^ was used as an acceptable cancer risk, and cancer risks greater than that value were assumed to have potential for causing carcinogenic diseases because the cancer risks of miners for occupational exposure to heavy metals in polluted soil were considered in this study. Unlike the reference dose for noncarcinogenic health risk assumes, the cancer slope factor (CSF) that exposures to any amount of a carcinogen will produce the cancer risk, i.e., there is no threshold dosage. A cancer slope factor is an upper bound, approximating a 95% confidence limit, on the increased cancer risk from lifetime exposure to toxicants by the dermal exposure route^73,74^. All methods in this study were performed in accordance with the relevant guidelines and regulations.

## Results

### Skin surface area of the miners

In this study, the total body surface areas of the miners were derived by using the weights and heights of 53 participants (42 male and 11 female) obtained from a questionnaire survey. The results showed that the height of all the participants ranged from 146.3 cm to 179.8 cm, with a median of 149.4 cm and a mean height (± SD) of 163.2 (± 8.8) cm. For male respondents, height ranged from 155.5 cm to 179.8 cm, and the mean height (± SD) was 166.8 (± 5.9) cm, whereas height of the female participants ranged from 146.3 cm to 152.4 cm, with a mean (± SD) of 149.4 (± 1.8) cm. The body weight of all the participants ranged from 46 to 75 kg, the median was 59 kg, and the mean (± SD) was 58.72 (± 6.8) kg. Ranging from 52 to 75 kg with a mean (± SD) weight of 60.76 (± 5.8) kg and ranging from 46 to 60 kg with a mean (± SD) weight of 50.9 (± 9.6) kg were for male and female participants, respectively. The body mass index of the participants was measured based on their heights and weights obtained from the questionnaire by using the formula body weight (kg)/height 2 (m), most of the participants (92.5%) were in the normal range (18.5–24.9), and only 7.5% of the participants were overweight (25–29.9). By using the average weight and height of the male and female participants, the average total body surface area of the miners in the study area was calculated through Eq. (2), which is mentioned in “Exposure assessment”, and Supplementary Table 3 shows the average total body surface area in meters of male and female participants.

### Job responsibilities of the participants in the gold mining processes

Regarding the main responsibilities of the participants, most of them (47%) in this study mainly worked in the ore processing step called hydraulic mining and dredging. The other 27% of the participants worked in the sluicing step; 15% of them worked in the panning step; and 11% of them were working in the amalgamation step. Panning and amalgamation steps are generally conducted by female miners. However, the miners sometimes had to be involved in the other gold mining steps that were not their main responsibilities because of the requirements of the job. Furthermore, the numbers of miners might be expanded or diminished depending on the accessibility of gold, and the highest numbers of miners are generally utilized in the ore processing step followed by sluicing. Supplementary Table 4 shows the main job responsibilities of the participants regarding the numbers of miners in each gold mining step of the mining sites. In addition, the mean (± SD) exposure frequency for all miners was 331 (± 51) days per year and ranged from 209 to 365 days, and the mean (± SD) exposure duration was 1.51 (± 0.77) years and ranged from 1 to 4 years as shown in supplementary table 5.

### PPE use behaviors of the miners

Although the miners could be exposed to the soil on their body parts while working, 42 participants (79%) did not use any form of personal protective equipment (PPE) among 53 participants. However, 11 participants (21%) used more than one type of PPE. Among them, 5 participants (9%) used hand-length fabric gloves as shown in Supplementary Fig. 1, 4 participants (8%) used hand-length rubber gloves as shown in Supplementary Fig. 2, and 3 participants (6%) used mid-length rubber boots as shown in Supplementary Fig. 3, in which one participant wore both hand-length rubber gloves and mid-length rubber boots. Correspondingly, another study in the Upper East Region in Ghana showed that 70% out of 120 miners did not use any form of PPE^75^. Nonetheless, some of the participants, including those who did not use any PPE or who used PPE, wore more than one type of normal clothing while working. Fifty-one percent of the participants used full trousers, 17% of them knee-length pants, 87% of them used long-sleeve shirts, 8% of them used elbow-length shirts, and 8% of them used shorter-than-elbow-length shirts. These kinds of normal clothes and fabric gloves might not protect against heavy metal exposure on miners’ body parts during work. Moreover, using only hand-length rubber gloves and mid-length rubber boots cannot provide full protection of the whole body from heavy metal exposure.

### Concentrations of heavy metals and soil pH in the soil samples from the gold mining sites

Table 1 shows the concentrations of the heavy metals and soil pH for the gold mining sites in terms of each small-scale gold mining step.

**Table 1:** Concentrations of the heavy metals in soils and soil pH.

Gold mining steps	As Mean ± SD (mg/kg)	Cd Mean ± SD (mg/kg)	Hg Mean ± SD (mg/kg)	Pb Mean ± SD (mg/kg)	pH Mean ± SD
**Ore processing**					
Site A	5.69 ± 0.14	0.27 ± 0.01	0.47 ± 0.23	9.73 ± 0.64	7.43 ± 0.42
Site B	6.55 ± 1.25	0.27 ± 0.02	0.68 ± 0.43	11.80 ± 1.73	5.91 ± 0.35
Site C	1.80 ± 0.48	0.33 ± 0.04	0.53 ± 0.13	12.80 ± 2.99	8.57 ± 0.11
Average ± SD	4.68 ± 2.53	0.29 ± 0.03	0.56 ± 0.11	11.44 ± 1.57	7.30 ± 1.33
**Sluicing**					
Site A	1.04 ± 0.19	0.36 ± 0.02	0.15 ± 0.03	8.60 ± 1.56	8.54 ± 0.22
Site B	9.02 ± 5.60	0.40 ± 0.12	0.35 ± 0.11	10.27 ± 3.50	6.91 ± 0.58
Site C	1.31 ± 0.22	0.13 ± 0.06	0.51 ± 0.04	7.67 ± 0.90	8.45 ± 0.10
Average ± SD	3.79 ± 4.53	0.30 ± 0.15	0.34 ± 0.18	8.85 ± 1.32	7.97 ± 0.92
**Panning**					
Site A	1.24 ± 0.42	1.32 ± 0.16	4.86 ± 5.87	34.40 ± 13.22	7.73 ± 0.15
Site B	16.25 ± 2.29	1.67 ± 0.89	1.53 ± 0.49	74.67 ± 65.62	7.70 ± 0.18
Site C	14.89 ± 6.97	0.61 ± 0.36	0.58 ± 0.31	27.33 ± 4.16	7.95 ± 0.50
Average ± SD	10.79 ± 8.30	1.20 ± 0.54	2.32 ± 2.25	45.47 ± 25.54	7.79 ± 0.14
**Amalgamation**					
Site A	15.58 ± 11.98	3.03 ± 0.47	40.95 ± 5.81	75.67 ± 25.01	4.87 ± 2.12
Site B	20.72 ± 7.38	3.07 ± 0.35	77.44 ± 16.73	210.00 ± 17.32	7.78 ± 0.10
Site C	22.17 ± 12.58	1.13 ± 0.75	35.73 ± 1.88	39.33 ± 7.51	7.39 ± 0.06
Average ± SD	19.49 ± 3.46	2.41 ± 1.11	51.37 ± 22.72	108.33 ± 89.90	6.68 ± 1.58

In this study, the concentration of As ranged from 1.04 to 22.17 mg kg^−1^; Cd ranged from 0.13 to 3.07 mg kg^−1^; Hg ranged from 0.15 to 77.44 mg kg^−1^; and Pb ranged from 7.67 to 210.00 mg kg^−1^. The consideration of each gold mining site, the concentrations of As at Site A were ranged from 1.04 to 15.58 mg kg^−1^, Cd ranged from 0.27 to 3.03 mg kg^−1^, Hg ranged from 0.15 to 40.95 mg kg^−1^, and Pb ranged from 8.60 to 75.67 mg kg^−1^. At site B, the concentrations of As were ranged from 6.55 to 20.72 mg kg^−1^, Cd ranged from 0.27 to 3.07 mg kg^−1^, Hg ranged from 0.35 to 77.44 mg kg^−1^, and Pb ranged from 10.27 to 210.00 mg kg^−1^. At site C, the concentrations of As were ranged from 1.31 to 22.17 mg kg^−1^, Cd ranged from 0.13 to 1.13 mg kg^−1^, Hg ranged from 0.51 to 35.73 mg kg^-1^, and Pb ranged from 7.67 to 39.33 mg kg^−1^.

By means of each heavy metal compared in each gold mining site, the order of concentrations could be shown for each gold mining step. In the ore processing step, the order was Cd > Hg > As > Pb at Site A, Cd > Hg > As > Pb at Site B, and Cd > Hg > As > Pb at Site C. In the sluicing step, the order was Hg > Cd > As > Pb at Site A, Hg > Cd > As > Pb at Site B, and Cd > Hg > As > Pb at Site C. In the panning step, the order was As > Cd > Hg > Pb at Site A, Hg > Cd > As > Pb at Site B and Site C similarly. In the amalgamation step, all the mining sites were similar order that was Cd > As > Hg > Pb.

In terms of each mining steps comparing, at site A, the order was sluicing > panning > ore processing > amalgamation for As, ore processing > sluicing > panning > amalgamation for Cd, and sluicing > ore processing > panning > amalgamation similar for Hg and Pb. At Site B, the order for As and Cd were similar as ore processing > sluicing > panning > amalgamation, for Hg and Pb were similar as sluicing > ore processing > panning > amalgamation. At site C, all heavy metals were similar as sluicing > ore processing > panning > amalgamation.

For the gold mining sites comparing, the concentrations of As in ore processing was Site C > Site A > Site B, in sluicing steps was Site A > Site C > Site B, in panning steps was Site A > Site C > Site B, and in amalgamation steps was Site A > Site B > Site C. For the Cd concentrations, in ore processing steps was Site A > Site B > Site C, in sluicing steps was Site C > Site A > Site B, in panning and amalgamation steps were similar order as Site C > Site A > Site B. For Hg concentration, in ore processing steps was Site A > Site C > Site B, in sluicing steps was Site A > Site B > Site C, in panning steps was Site C > Site B > Site A, and in amalgamation steps was Site C > Site A > Site B. For Pb concentration, in ore processing steps was Site B > Site A > Site C, in sluicing, panning and amalgamation steps were similar order as Site C > Site A > Site B.

In the case of the soil pH, the results revealed that very strongly acidic, moderately acidic, neutral, slightly alkaline, moderately alkaline and strongly alkaline soil pH values were found in this study. However, very strongly acidic (pH = 4.87) soil was found only in the amalgamation step of Site A; moderately acidic (pH = 5.91) soil was found only in the ore processing step of Site B; and neutral (pH = 6.91) soil was found only in the sluicing step of Site B. In the other steps of all the gold mining sites, all the pH values were alkaline, and the pH of these gold mining steps varied from slightly alkaline (pH = 7.39) to strongly alkaline (pH = 8.57). More specifically, the pH in the gold mining steps of site A ranged from 4.87 to 8.54, that of site B ranged from 5.91 to 7.78, and that of site C ranged from 7.39 to 8.57.

### Non-carcinogenic risks and carcinogenic risks of the heavy metals for dermal exposure

To calculate noncancer risks for the miners, the ADI for the dermal exposure route was calculated by Eq. (1), using the exposure parameters presented in Tables 1, 2 and 3. Then, hazard quotients were calculated based on the RfD values by using Eq. (3), and then hazard indices were calculated using Eqs. (4) and (6).

**Table 2:** Non-carcinogenic risks for the miners in site A, B, and C.

Heavy metals	Ore processing		Sluicing		Panning		Amalgamation	
	Male	Female	Male	Female	Male	Female	Male	Female
**Hazard quotient (HQ) site A**								
As	4.30 × 10^–2^	4.46 × 10^–2^	7.85 × 10^–3^	8.14 × 10^–3^	9.36 × 10^–3^	9.71 × 10^–3^	1.18 × 10^–1^	1.22 × 10^–1^
Cd	6.10 × 10^–4^	6.35 × 10^–4^	8.15 × 10^–4^	8.45 × 10^–4^	2.99 × 10^–3^	3.10 × 10^–3^	6.85 × 10^–3^	7.10 × 10^–3^
Hg	1.52 × 10^–3^	1.58 × 10^–3^	4.84 × 10^–4^	5.01 × 10^–4^	1.57 × 10^–2^	1.63 × 10^–2^	1.32 × 10^–1^	1.38 × 10^–1^
Pb	6.12 × 10^–3^	6.35 × 10^–3^	5.41 × 10^–3^	5.61 × 10^–3^	2.16 × 10^–2^	2.25 × 10^–2^	4.76 × 10^–2^	4.94 × 10^–2^
HI	5.13 × 10^–2^	5.32 × 10^–2^	1.46 × 10^–2^	1.51 × 10^–2^	4.97 × 10^–2^	5.16 × 10^–2^	3.04 × 10^–1^	3.17 × 10^–1^
**Hazard quotient (HQ) site B**								
As	4.95 × 10^–2^	5.13 × 10^–2^	6.81 × 10^–2^	7.06 × 10^–2^	1.23 × 10^–1^	1.27 × 10^–1^	1.56 × 10^–1^	1.62 × 10^–1^
Cd	6.10 × 10^–4^	6.35 × 10^–4^	9.05 × 10^–4^	9.40 × 10^–4^	3.79 × 10^–3^	3.93 × 10^–3^	6.95 × 10^–3^	7.20 × 10^–3^
Hg	2.20 × 10^–3^	2.28 × 10^–3^	1.13 × 10^–3^	1.17 × 10^–3^	4.97 × 10^–3^	5.14 × 10^–3^	2.51 × 10^–1^	2.60 × 10^–1^
Pb	7.42 × 10^–3^	7.70 × 10^–3^	6.46 × 10^–3^	6.70 × 10^–3^	4.70 × 10^–2^	4.87 × 10^–2^	1.32 × 10^–1^	1.37 × 10^–1^
HI	5.97 × 10^–2^	6.19 × 10^–2^	7.66 × 10^–2^	7.94 × 10^–2^	1.79 × 10^–1^	1.85 × 10^–1^	5.46 × 10^–1^	5.66 × 10^–1^
**Hazard quotient (HQ) site C**								
As	1.36 × 10^–2^	1.41 × 10^–2^	9.89 × 10^–3^	1.03 × 10^–2^	1.12 × 10^–1^	1.17 × 10^–1^	1.67 × 10^–1^	1.74 × 10^–1^
Cd	7.50 × 10^–4^	7.75 × 10^–4^	2.95 × 10^–4^	3.10 × 10^–4^	1.38 × 10^–3^	1.44 × 10^–3^	2.56 × 10^–3^	2.66 × 10^–3^
Hg	1.71 × 10^–3^	1.78 × 10^–3^	1.65 × 10^–3^	1.71 × 10^–3^	1.88 × 10^–3^	1.95 × 10^–3^	1.16 × 10^–1^	1.20 × 10^–1^
Pb	8.05 × 10^–3^	8.35 × 10^–3^	4.83 × 10^–3^	5.01 × 10^–3^	1.72 × 10^–2^	1.78 × 10^–2^	2.47 × 10^–2^	2.57 × 10^–2^
HI	2.41 × 10^–2^	2.50 × 10^–2^	1.67 × 10^–2^	1.73 × 10^–2^	1.32 × 10^–1^	1.38 × 10^–1^	3.10 × 10^–1^	3.22 × 10^–1^

**Table 3:** Carcinogenic risks of the miners for exposure to As in soil for site A, B and C.

Cancer risks	Ore processing					Sluicing						Panning						Amalgamation					
	Male		Female			Male			Female			Male			Female			Male				Female	
**Site A**																							
Current	4.49 × 10^–7^		4.39 × 10^–7^			8.21 × 10^–8^			8.02 × 10^–8^			9.79 × 10^–8^			9.56 × 10^–8^			1.23 × 10^–6^				1.20 × 10^–6^	
Estimated for 9 years	2.68 × ^–6^10		2.62 × 10^–6^			4.89 × 10^–7^			4.78 × 10^–7^			5.83 × 10^–7^			5.70 × 10^–7^			7.33 × 10^–6^				7.16 × 10^–6^	
**Site B**																							
Current	5.17 × 10^–7^		5.05 × 10^–7^			7.12 × 10^–7^			6.96 × 10^–7^			1.28 × 10^–6^			1.25 × 10^–6^			1.64 × 10^–6^				1.60 × 10^–6^	
Estimated for 9 years	3.08 × 10^–6^		3.01 × 10^–6^			4.24 × 10^–6^			4.15 × 10^–6^			7.65 × 10^–6^			7.47 × 10^–6^			9.75 × 10^–6^				9.52 × 10^–6^	
**Site C**																							
Current	1.42 × 10^–7^		1.39 × 10^–7^			1.03 × 10^–7^			1.01 × 10^–7^			1.18 × 10^–6^			1.15 × 10^–6^			1.75 × 10^–6^				1.71 × 10^–6^	
Estimated for 9 years	8.47 × 10^–7^		8.27 × 10^–7^			6.16 × 10^–7^			6.02 × 10^–7^			7.01 × 10^–6^			6.84 × 10^–6^			1.04 × 10^–5^				1.02 × 10^–5^	

The detailed results of HI for both male and female miners from Site A, Site B and Site C exposed to As, Cd, Hg, and Pb through dermal exposure to the soil are presented in Table 2, respectively. At Site A, the HI results for the miners in all gold mining steps ranged from 1.46 × 10^–2^ to 3.17 × 10^–1^. At Site B, The HI results ranged from 5.97 × 10^–2^ to 5.66 × 10^–1^. At Site C, the HI results ranged from 1.67 × 10^–2^ to 3.22 × 10^–1^. The lowest HI value was found as 1.46 × 10^–2^ in the sluicing step at Site A, and the highest HI value was found as 5.66 × 10^–1^ in the amalgamation step at Site B. Therefore, all the HI results for the miners from the three mining sites are less than 1, which indicates that all the participants in this study might not be suffering from any noncancerous diseases.

In case of carcinogenic risks, only As is likely to be carcinogenic through dermal contact with the soil among the heavy metals listed in this study according to U.S. EPA and the International Agency Research on Cancer (IARC). Therefore, the carcinogenic risk of As for the miners from three mining sites was calculated through Eq. (1) for ADI and Eq. (5) for cancer risks assessment by using the exposure parameters and cancer slope factor from “Dose-response assessment”. In this study, two kinds of cancer risks calculations were conducted by using two different values of exposure duration. In the first calculation, the current cancer risk of the miners from the three mining sites were estimated by assuming their average current years of working in their respective mining sites obtained from questionnaire survey as exposure duration. The second calculation was carried out by using the central tendency exposure duration that is recommended from U.S. EPA in order to estimate the cancer risks of the miners for 9 years. The result of the cancers risks of the miners from Site A, Site B and Site C are shown in Table 3, respectively.

Regarding the current cancer risk assessment of the miners, the results ranged 8.02 × 10^–8^ to 1.23 × 10^–6^ in Site A; 5.05 × 10^–7^ to 1.64 × 10^–6^ in Site B; and 1.01 × 10^–7^ to 1.75 × 10^–6^ in Site C. The lowest current cancer risk was found as 8.02 × 10^–8^ in the sluicing step of Site A, and the highest current cancer risk was found as 1.75 × 10^–6^ in the amalgamation step of Site C. Therefore, the results that are greater than acceptable U.S. EPA cancer risks level, 1 × 10^–6^ (i.e., 1 case of cancer per every 1,000,000) were investigated in the step of amalgamation in all the entire mining sites and in the step of panning in Site B and Site C. Thus, it indicates that the miners in this study may have the potential to suffer the symptoms of As related carcinogenic diseases even with the current working experience.

In case of the estimated cancer risk for 9 years, the results ranged 4.78 × 10^–7^ to 7.33 × 10^–6^ in Site A; 3.01 × 10^–6^ to 9.75 × 10^–6^ in Site B; and 6.02 × 10^–7^ to 1.04 × 10^–5^ in Site C. The lowest risk was found as 4.78 × 10^–7^ in the sluicing step of Site A, and the highest risk was found as 1.04 × 10^–5^ in the amalgamation step of Site C. Therefore, the risks above the acceptable U.S. EPA cancer risks level were found in the amalgamation steps of all mining sites, panning steps of Site B and Site C, sluicing step of Site B and ore processing steps of Site A and Site B. Therefore, it indicates that the miners in this study may have As related cancerous diseases if they continue working up to 9 years in the same mining site or other mining sites that have the similar concentration level of As without using any personal protective equipment (PPE).

## Discussion

### Concentrations of heavy metals and ph levels in the soil in different gold mining steps

The concentrations of heavy metals among the gold mining sites can be varied by depending on mineralogy, exploration types, technology, and beneficiation techniques. Among the gold mining sites in this study, the highest concentrations of As (22.17 mg kg^−1^) was found in the amalgamation step at Site C. In the amalgamation steps, the concentrations of As at Site A, Site B and Site C were not significant different. However, As concentrations in the panning step at Site A was lower than As at Site B and Site C. It could be explained by considering about water used for the mining process. At Site A, panning step was not conducted in the mine site while the other gold mining steps were conducted in. In the panning step at Site A, the cancer risk was found to be in the acceptable range because of the relatively low As concentration, explained by considering the water used for the mining process. At Site A, miners carried gold-containing waterproof carpets via trucks to a specific place in a village that was approximately 10 km from the mining site for panning. Then, water from a stream near the village was used for panning. The concentration of As in the stream water was assumed to be much lower than the concentration of As in the water in the mining area. Thus, the concentration of As in the soil samples of the panning step at Site A was very low compared with the concentrations at the other sites. In addition, the concentration of As in the amalgamation step of Site A was relatively higher than the concentration of As in the panning step since the amalgamation step was conducted at the mining site by using water from the mining area. Therefore, the concentrations of heavy metals in the soil of the gold mining steps can vary depending on the water source used in the mining process.

The highest As concentration in this study is also radically lower than As concentrations of 591 mg kg^−1^ in the soil of ASGM area in Philippines^76^. Moreover, it is also extremely lower than the recently reported amount of As concentration (1560 to 10,000 mg kg^−1^) in the tailing of gold mining area in eastern Amazon^77^. Therefore, the less As concentrations in the study area may be related to the existence of low As-rich minerals. In the case of Cd, the highest concentration was found at Site B at 3.07 mg kg^−1^ that was less than the previous studies in the soil of mining area in Tanzania (6.40–11.70 mg kg^−1^)^36^ and in China (11.7 mg kg^−1^)^78^, but it was higher than the study in South Africa (0.05 mg kg^−1^)^47^. The highest concentration of Hg was found at Site B (77.44 mg kg^−1^) that was higher than the average Hg soil concentration of 71 mg kg^−1^ in the soil of gold mining community in Ghana^79^ and in South Africa (0.090 mg kg^−1^)^47^. However, the results of the study was drastically lower than the Hg soil concentration of previous studies in Iran (100 mg kg^−1^)^39^ and Kenya (1920 mg kg^−1^)^37^. The highest concentration of Pb in this study was investigated at Site B (210 mg kg^−1^). In this study, Pb concentrations are greater than all the other heavy metals concentrations in all the gold mining steps of each mining site. It might be the elevated concentration Pb in the ore origin of study sites as Pb is gold associated heavy metal. Similarly, previous studies reported high concentrations of Pb in the soil of gold mining areas in Kenya (510 mg kg^−1^)^42^ and Philippines (6370 mg kg^−1^)^76^. However, the results in this study is about 42 times higher than that of concentration in South Africa (4.790 mg kg^−1^)^47^ and about 3 times greater than the previous study in Oman (80 mg kg^−1^)^38^.

In this study, the highest concentrations of all the heavy metals were investigated at the amalgamation steps in all gold mining sites, explained by considering gold recovery through the small-scale gold mining technique. In the small-scale gold mining technique, gold is recovered through the gravity method such as sluicing and panning as the density of gold (19.39 g cm^−3^) is higher than the density of other minerals in the ore. Consequently, the largest mass of gold is attained in the amalgamation step among the other gold mining steps: ore processing, sluicing and panning. Similarly, the density of the heavy metals: As (5.7 g cm^−3^), Cd (8.7 g cm^−3^), Hg (13.6 g cm^−3^) and Pb (11.34 g cm^−3^) is also higher than the density of other minerals in the ore^80^. Therefore, the highest concentrations of all the heavy metals together with the largest mass of gold could be found in the amalgamation step. In addition, significantly higher concentrations of Hg were also found in the amalgamation all three gold mining sites because Hg is used mainly to form a stable amalgam to extract gold from gold ore in the amalgamation step; thereby releasing Hg into nearby water, soil, sediment and even into the atmosphere through the process of burning the gold-Hg amalgam. As a consequence, Hg can cause contamination of the atmosphere, soil, underground water, rivers, and lakes, and eventually, the bioconcentration of methylmercury is contributed by the process of methylation through bacteria and food chains, thereby leading to critical environmental issues and health problems.

In the case of soil pH, strongly alkaline was investigated in Site C as 8.57 in this study. Similarly, the high soil pH level (alkalinity) was also reported as 8.80 in the soil of the artisanal gold mining area in northern Atbara, Sudan^81^. However, strongly acidity the soil pH level in the amalgamation step of Site A was found to be strongly acidic at 4.87. Similar soil pH results were reported as 4.6–5.4 in the study of chemical and mineralogical properties in two gold mining sites in Ghana^82^. Likewise, another study also reported soil pH values of 2–6.23 in gold mine tailings in South Africa^48^. Regarding the acidic soil pH level, Site A could therefore be considered to have refined gold through acid digestion (aqua regia digestion) near the place of amalgamation. Otherwise, it could also be considered that the cyanide leaching technique is being used to further recover gold from Hg-rich soil attained from the amalgamation step. In the cyanide leaching technique, mercury-rich soil is processed in a tank by adding sodium cyanide (NaCN), which solubilizes gold^83^. After the completion of solubilization, active carbon was used to recover gold by absorbing gold onto the surface of active carbon from the complex solution of gold-cyanide^84^. Finally, acid residues from the process of stripping gold from the active carbon can cause a decrease in soil acidity^85^.

### Non-carcinogenic and carcinogenic risks of the heavy metals

In this study, the result of HI for all the gold mining sites ranged from 1.46 × 10^–2^ to 5.66 × 10^–1^ and all the miners in the study sites may not have any non-cancerous diseases. This study found HQ of As ranging from 7.85 × 10^–3^ to 1.74 × 10^–1^. The similar results of HQ for As were also reported in the previous studies in the gold mining areas: HQ value of 7.07 × 10^–2^ in Igun, Osun State, Nigeria^45^ and 5.90 × 10^–2^ in Krugersdorp, South Africa^48^. Unlikely, unacceptable HQ result of 1.3 for As was reported in the gold mining operation in Bolivian Andes^86^, and very high non-cancer risk was also reported in the eastern Amazon^77^. HQ of Cd in this study ranged from 2.95 × 10^–4^ to 7.20 × 10^–3^. Similarly, HQ of Cd was reported as 2.34 × 10^–3^ in Nigeria^45^, and the greater HQ value of Cd was reported as 5.10 × 10^–1^ in South Africa^48^. In case of Hg, HQ results ranged 4.84 × 10^–4^ to 2.60 × 10^–1^ in this study that is similar with the study in Bolivian Andes (7.70 × 10^–2^)^86^. HQ results for Pb in this study ranged from 4.83 × 10^–3^ to 1.37 × 10^–1^, and the results are also in line with the study in Nigeria (7.76 × 10^–4^)^45^, and South Africa (3.40 × 10^–2^).

For the cancer risks, the highest current cancer risk was 1.75 × 10^–6^, and the highest estimated cancer risk for 9 years was 1.04 × 10^–5^. Similar result of cancer risk (2.4 × 10^–5^) for the dermal exposure to As was reported in the study of gold mining operation in Bolivian Andes^86^. Likewise, the other previous studies in gold mining areas from different countries were also reported lifetime cancer risks for dermal exposure to As in the soil. The cancer risks of 7.7 × 10^–6^ was reported in China^43^, 1.73 × 10^–5^ was reported in Witwatersrand gold mining basin, South Africa^47^, and 2.2 × 10^–3^ was reported in Krugersdorp, South Africa^48^. A recent study also reported very high cancer risk of As in the gold mining areas in the eastern Amazon^77^. Unlikely, even the current cancer risk of this study is higher than the lifetime cancer risk for dermal exposure (9.46 × 10^–8^) of a study in artisanal gold mines of Nigeria^87^. Therefore, PPE is suggested to be a protective factor of heavy metal exposure in occupational risk^88^.

In this study, all the highest concentrations, HI value and cancer risks were found in the amalgamation steps of all gold mining sites. By considering the job responsibilities, almost all of the miners in the amalgamation steps were women of childbearing age. Therefore, female miners in this step may have very high potential to transmit the heavy metals especially Hg to the fetus in utero and to infants through breast milk^1,89^. Even male miners can also be carriers of heavy metals to their children through body contact and body to objects as children have the behavior of hand-to-mouth movement.

Moreover, there are no proper management and waterproofing agents for the waste deposits in all the mining sites in this study. Solubilization and leaching of chemical may also be occurred as the tailings are formed by sand-sized particles^77^. Therefore, heavy metals from the tailings may be released into the nearby environment through rainfall. Consequently, communities may be exposed to the heavy metal from mining waste through different pathways, and then it may lead to suffer potential cancer and non-cancer diseases.

The concentrations of soil heavy metals can vary by depending physical and chemical properties of soil^90,91^, only soil pH was measured in this study. The limited of this study was, this study did not conduct assessing enrichment factor (EF), contamination factor (CF) and pollution load index (PLI) that can investigate pollution status of heavy metals in soil^91,92^. The noncancer and cancer risk assumptions in this study could lead to overestimation if the concentration levels of the listed heavy metals in the soil decline. Conversely, the risk assumptions could also lead to underestimation if mining activities are increased. Furthermore, exposure to the listed heavy metals in other media such as air, water and food was not considered in this study. Therefore, exposure to the listed heavy metals in other media could also lead to underestimation of the risks. However, this type of study provides baseline information for future studies on such public health concerns as pollutants and policy interventions.

## Conclusions

The gold mining process can cause the health risk to miners from dermal exposure, this study found panning and amalgamation steps have the most potential to cause carcinogenic risk. All hazard quotient and hazard index were lower than acceptable level (HQ < 1, HI < 1). In addition, the highest noncancer risk was found as 5.66 × 10^–1^ and cancer risks as 1.04 × 10^–5^ were investigated in the amalgamation process. The significant Hg concentrations were found in the amalgamation steps of all the gold mining sites. Although the noncancer risk was not found, but the results of cancer risk revealed that the small-scale gold miners are at high risk cause the dermal exposure to As. In fact, there is not only four heavy metals such as As, Cd, Hg, and Pb that contaminated in mining soil, but might also be found other heavy metal. Thus, miners may also be at risks of cancer and noncancer diseases because of exposure to the other heavy metal and might from other exposure route such as through inhalation and ingestion pathways. Moreover, according to the behavior of minor result, this study would like to suggest that to educate miners about the health risks and encourage them using proper PPE all the time while working in the gold mine can prevent adverse health effects and suffering from heavy metals exposure in the future. This study can be a guideline and significant information for the occupational health risks of gold miners regarding to heavy metals exposure. Not only affected to human but Hg can be released into the nearby environment, and it can finally cause serious environmental problems. Therefore, the further studies of heavy metals release from mining to surrounding and health risk to community nearby should be considered as well.

## Supplementary Information

Supplementary Information.

## Data Availability

Soil samples were collected through a steel trowel from 3 small-scale gold mining sites from Banmauk Township, Myanmar. The required materials for soil sample collection were supported by the Center of Excellence on Hazardous Substance and Environmental Management, Chulalongkorn University, Thailand.
