# Supplementary material for: Related health risk assessment of exposure to arsenic and some heavy metals in gold mines in Banmauk Township, Myanmar
Source: Sci Rep. 2021 Nov 24;11:22843. doi: 10.1038/s41598-021-02171-9 (PMC8613182; doi:10.1038/s41598-021-02171-9)
Supplement: Supplementary file 1 — Supplementary Information. [file 41598_2021_2171_MOESM1_ESM.docx]

**Supplementary Table 1** Limit of detection (LOD), limit of quantification (LOQ), coefficient of determination (r^2^), and relative standard deviation (RSD)

| **Metal** | **LOD (mg/kg)** | **LOQ (mg/kg)** | **Coefficient of determination (r^2^)** | **RSD%** |
| --- | --- | --- | --- | --- |
| As | 0.0007 | 0.0023 | ≥ 0.999 | ≤ 5% |
| Cd | 0.0099 | 0.0330 | ≥ 0.999 | ≤ 5% |
| Hg | 0.0003 | 0.0010 | ≥ 0.999 | ≤ 5% |
| Pb | 0.0690 | 0.2300 | ≥ 0.999 | ≤ 5% |

**Supplementary Table 2** Exposure Parameters for dermal exposure (ADI_dermal_)

| **Parameter** | **Symbol** | **Unit** | **Adult** | | **References** |
| --- | --- | --- | --- | --- | --- |
|  |  |  | U.S.EPA Default Value | Questionnaire |  |
| Skin Surface Area | SA | cm^2^/day | - | * |  |
| Soil Adherence factor | AF | mg/cm^2^ | 0.07 | - | (USEPA, 2017) |
| Conversion factor | CF | kg/mg | 10^-6^ | - | (USEPA, 1989) |
| Absorption factor | ABS | unitless | 0.13 | - | (USEPA, 2017) |
| Exposure frequency | EF | days/yr | - | * |  |
| Exposure duration | ED | yrs | 9 | * | (USEPA, 2017) |
| Averaging time | AT | days |  |  |  |
| For carcinogens | | days | 365 x life expectancy |  | (USEPA, 2017) |
| For non-carcinogens | | days | 365 x ED |  | (USEPA, 2017) |
| Life expectancy in Myanmar: Male = 65; Female = 69; Average = 67 | | | | | (WHO, 2018) |

*Data were obtained from questionnaire.

**Supplementary Table 3** Average total body surface area of the miners (m^2^)

|  | Weight (kg)  Mean ± SD | Height (cm)  Mean ± SD | Total body surface area (m^2^)  Mean ± SD |
| --- | --- | --- | --- |
| Male (n = 42) | 60.76 ± 5.81 | 166.77 ± 5.89 | 1.68 ± 0.10 |
| Female (n = 11) | 50.91 ± 3.87 | 149.35 ± 1.84 | 1.46 ± 0.06 |

**Supplementary Table 4** Job responsibilities of the participants in the four gold mining steps

| **Gold mining sites** | **Numbers of miners (%) in the gold mining steps** | | | |
| --- | --- | --- | --- | --- |
|  | **Ore processing** | **Sluicing** | **Panning** | **Amalgamation** |
| Site A (n=19) | 8 (42%) | 5 (26%) | 3 (16%) | 3 (16%) |
| Site B (n=17) | 8 (47%) | 5 (29%) | 2 (12%) | 2 (12%) |
| Site C (n=17) | 9 (53%) | 4 (24%) | 3 (18%) | 1 (6%) |
| **Total (n=53)** | **25 (47%)** | **14 (27%)** | **8 (15%)** | **6 (11%)** |

**Supplementary Table 5** Average exposure frequency and exposure duration of the miners

| **Exposure parameters** | **Mean ± SD** |
| --- | --- |
| Exposure Frequency (EF) (day/year) | 331 ± 51 |
| Exposure Duration (ED) (year) | 1.51 ± 0.77 |

**Supplementary Figure 1** Hand-length fabric gloves use behavior of the participants

**Supplementary Figure 2** Hand-length rubber gloves use behavior of the participants

**Supplementary Figure 3** Mid-length rubber boots use behavior of the participants
